# A Novel and Label-Free Chemiluminescence Detection of Zearalenone Based on a Truncated Aptamer Conjugated with a G-Quadruplex DNAzyme

**DOI:** 10.3390/bios13010118

**Published:** 2023-01-09

**Authors:** Yue Guan, Junning Ma, Jing Neng, Bolei Yang, Yan Wang, Fuguo Xing

**Affiliations:** 1 College of Food Science and Technology, Zhejiang University of Technology, Hangzhou 310014, China; 2 Key Laboratory of Agro-Products Quality and Safety Control in Storage and Transport Process, Ministry of Agriculture and Rural Affairs, Beijing 100193, China; 3 Institute of Food Science and Technology, Chinese Academy of Agricultural Sciences, Beijing 100193, China

**Keywords:** chemiluminescence, label-free, aptasensor, DNAzyme, ZEN

## Abstract

Zearalenone (ZEN), one of the most frequently occurring mycotoxin contaminants in foods and feeds, poses considerable threat to human and animal health, owing to its acute and chronic toxicities. Thus, rapid and accurate detection of ZEN has attracted broad research interest. In this work, a novel and label-free chemiluminescence aptasensor based on a ZEN aptamer and a G-quadruplex DNAzyme was constructed. It was established on a competitive assay between ZEN and an auxiliary DNA for the aptamer, leading to activation of the G-quadruplex/hemin DNAzyme and subsequent signal amplification by chemiluminescence generation after substrate addition. To maximize the detection sensitivity, numerous key parameters including truncated aptamers were optimized with molecular docking analysis. Upon optimization, our aptasensor exhibited a perfect linear relationship (R^2^ = 0.9996) for ZEN detection in a concentration range of 1–100 ng/mL (3.14–314.10 nM) within 40 min, achieving a detection limit of 2.85 ng/mL (8.95 nM), which was a 6.7-fold improvement over that before optimization. Most importantly, the aptasensor obtained a satisfactory recovery rate of 92.84–137.27% and 84.90–124.24% for ZEN-spiked wheat and maize samples, respectively. Overall, our label-free chemiluminescence aptasensor displayed simplicity, sensitivity, specificity and practicality in real samples, indicating high application prospects in the food supply chain for rapid detection of ZEN.

## 1. Introduction

Zearalenone (ZEN), one of the naturally occurring mycotoxins, is a secondary metabolite produced by members of the genus *Fusarium* [1,2]. It has elicited particular attention due to its extensive contamination of cereals such as corn, barley, wheat and their by-products [3]. Since ZEN has toxic effects including reproductive toxicity, genotoxicity and cytotoxicity, direct consumption or accumulation through the food chain causes acute or chronic poisoning to the health of animals and human [4]. Given the toxicity and contamination status of ZEN, many countries and organizations have established regulations on the maximum residue level (MRL) of ZEN in various cereals and their by-products. In China, the MRL of ZEN in cereals and their products is 60 μg/kg [5]. European Union sets the MRL for ZEN to be 75 μg/kg in cereals and cereal products [6]. For the particularly sensitive group of infants and young children, the European Union has set a stricter limit of 20 μg/kg [7]. Therefore, it is imperative to develop an easy-to-use tool for rapid and sensitive detection of ZEN.

In order to control the content of ZEN in cereal products, various analytical methods for accurate and sensitive quantification of ZEN have been developed. These methods include thin layer chromatography (TLC) [8,9], gas chromatography-mass spectrometry (GC-MS) [10,11], liquid chromatography fluorescence (LC-FL) [12] and high performance liquid chromatography (HPLC) [13,14]. Even though chromatographic methods display high precision and sensitivity, the side effects such as high cost of reagents, expensive instrumentation, need for professional operators and tedious sample processing steps make these methods not suitable for rapid and on-site determination of ZEN [1,15,16]. In contrast, enzyme-linked immunosorbent assay (ELISA) displays the advantages of low cost, good specificity and trace detection [16,17]. However, it has a strong dependence on the use of susceptible and expensive antibodies as well as accompanying high labelling cost, which limits its application for onsite and large-scale screening analysis [7,18].

Aptamers, as an emerging type of recognition element with high selectivity, are artificial antibodies developed mainly through the in vitro selection process referred to as the systematic evolution of ligands by exponential enrichment (SELEX) [19]. Aptamers can bind various targets with high specificity and affinity owing to their unique three-dimensional folding structures (hairpins, pseudoknots or bulges) in the presence of targets [20,21]. Aptamers are essentially single-stranded DNA or RNA, possessing excellent properties such as easy chemical modification, high thermal and chemical stability, low immunogenicity, long shelf life, and low toxicity [22,23,24]. They have been extensively integrated into the construction of biosensors for various targets, such as cancer markers [25], pesticides [26], ATP [27], antibiotics [28] and mycotoxins [29]. The binding modes between aptamers and targets include hydrogen bonding, van der Waals forces, π–π stacking, electrostatic interactions and so on [21]. Elucidation of the binding interactions between aptamers and targets with computational simulation or molecular docking provides the theoretical basis for aptamer optimization [21]. Optimization of aptamers using site-directed mutagenesis and rational truncation based on the aptamer–target binding mechanism can further boost aptamers’ binding affinity or biosensors’ performance [21,30,31,32]. Trinh et al. [32] truncated a SELEX-identified aptamer from 57 to 35 bases in the non-functional stem region and significantly increased its binding affinity to fipronil. In addition, site-directed mutagenesis is another effective approach for aptamer optimization. A single-point mutation from T to G in the loop region of a saxitoxin-binding aptamer increases its binding affinity more than fivefold, whereas multiple mutations from G to A in the loop of an aptamer compromises its binding affinity to fipronil and thioflavin [32,33]. Thus, truncation or site-directed mutation in aptamers should be carefully performed based on the accurately predicted interactions between aptamers and targets from molecular docking analysis in combination with validation using circular dichroism spectroscopy or nuclear magnetic resonance [21].

Similar to aptamers, catalytic nucleic acids (or DNAzymes) have attracted growing interests in recent years as they can be used for signal amplification in biosensing platforms by replacing traditional protein enzyme labels, due to their advantages of cheap synthesis and high stability [34]. DNAzyme can form hemin/G-quadruplex to facilitate H_2_O_2_-mediated oxidation of substrates to yield colorimetric or chemiluminescent signal [35,36] or can act as an RNA-cleaving DNAzyme to separate fluorophore from quencher moieties to enable a fluorescence signal [37]. Some metal ions such as K^+^ or Zn**^2^**^+^ can further enhance the catalytic activity of DNAzymes [38,39]. Based on this property, DNAzymes have been employed for detection of metal ions [37,40], mycotoxins [36,41] and pathogens [42] in combination with aptamers or recognition elements.

The combination of aptamers with DNAzymes can be used to fabricate diverse forms of biosensors through noncovalent or covalent bonding. The noncovalent bonding is mainly due to double strand hybridization or triple helix formation between an aptamer and a DNAzyme [38,41]. Yet, this combination adds further complexity to the sensing system or requires strict buffer conditions to function. For example, triple helix formation needs slightly acidic buffer conditions [38], which might compromise the aptamer’s binding affinity. In comparison, covalent bonding by simply conjugating a DNAzyme to an aptamer to form an aptazyme can solve the above issues. The aptazyme possesses the potential to be integrated in a label-free biosensing system in which the DNAzyme activity is activated once the aptamer–target binding occurs. This system’s merits include quick response, simplicity, high sensing capability and selectivity [43,44]. However, there is no documentation of an aptazyme for the chemiluminescence detection of ZEN. In this work, we reported a label-free chemiluminescence system based on an aptazyme constituting a G-quadruplex DNAzyme and a truncated ZEN aptamer linked by triple adenines. The chemiluminescence platform was fabricated based on the competitive assay between the ZEN ligand and an auxiliary oligo (partially complementary to the aptazyme) for the aptazyme. In the presence of ZEN, it binds to the ZEN aptamer segment, promoting the formation of G-quadruplex/hemin DNAzyme. Upon addition of luminol substrate and H_2_O_2_ mediator, the system can generate a strong chemiluminescence signal measurable with a microplate reader or other portable device such as a smartphone. The proposed method has no labelling cost, a short testing time, high sensitivity and specificity. Moreover, the proposed method has achieved a satisfactory result in the detection of ZEN in real cereal samples, thus exhibiting a broad application potential for ZEN detection in foodstuffs.

## 2. Materials and Methods

### 2.1. Chemicals and Reagents

N, N-dimethylformamide (DMF) was purchased from Aladdin Chemical Co. Ltd. (Shanghai, China). Hemin, luminol, 20× phosphate-buffered saline (pH 7.4), 1M Tris-HCL (pH 7.4) and 1M Tris-HCl (pH 9.0) were purchased from Solarbio Life Sciences (Beijing, China). The 1 mM hemin and 30 mM luminol were prepared in DMF and stocked in the dark at 4 °C. H_2_O_2_ (30%), methanol (HPLC grade), magnesium chloride hexahydrate and potassium chloride were obtained from Beijing Jingri Jindian Technology Co., Ltd. (Beijing, China). Zearalenone (ZEN), aflatoxin B_1_ (AFB_1_), deoxynivalenol (DON) and ochratoxin A (OTA) were ordered from Meizheng Bio-Tech (Beijing, China). Costar 96-well white flat-bottom microplates were purchased from Corning (Beijing, China). All the oligonucleotides were synthesized by Tsingke Biotechnology (Beijing, China) and prepared as stocks of 10 μM in nuclease-free water.

### 2.2. Apparatus

A Spark^®^ multimode microplate reader (Tecan Trading AG, Männedorf, Switzerland) was used for measurement of luminescence intensity. A thermostatic incubator and microtiter plate shaker for incubation of microplates were purchased from Jiangge Technology (Hangzhou, China).

### 2.3. Construction of Label-Free Chemiluminescence Aptasensor Based on DNAzyme–Aptamer Conjugate for ZEN Detection

All DNA oligonucleotides (Table S1 in Supplementary Materials) and ZEN were diluted using the ZEN binding buffer. They were heated to 95 °C for 5 min and cooled to room temperature just before use. To start, 70 μL 0.1 μM conjugate (DNAzyme_ZEN) of a DNAzyme [45] and a previously SELEX-identified ZEN aptamer [46] was mixed with 10 μL of ZEN solution in a 96-well microplate for affinity binding for 15 min at 25 °C. Subsequently, 10 μL of an axillary DNA oligo at 1 μM was added for competitive binding for 10 min at 25 °C. Following this, 10 μL of hemin solution was added for the assembly of the G-quadruplex/hemin DNAzyme at 25 °C for 10 min. Meanwhile, a chemiluminescence reaction mixture comprising 1 μL 30 mM luminol, 1 μL 200 mM H_2_O_2_ and 98 μL ZEN binding buffer was prepared and added for chemiluminescence generation. The luminescence intensity was immediately measured with a microplate reader.

For exploration of the suitability of our label-free chemiluminescence sensing system, four types of ZEN binding buffers and four hemin concentrations were probed. The ZEN binding buffers tested in this study were 1× PBS (pH 7.4), 10× PBS (pH 7.4), 100 mM Tris-HCl (pH 7.4) and 100 mM Tris-HCl (pH 9.0), with all buffers supplemented with 2.5 mM MgCl_2_ and 5 mM KCl for DNAzyme activity enhancement. Hemin concentrations of 500, 50, 5 and 0.5 μM were also tested for its impact on the feasibility of the aptasensor.

### 2.4. Molecular Docking between Truncated ZEN Aptamers and ZEN

The molecular docking procedures were implemented as per the method described previously [47,48]. Briefly, the secondary structure of DNA oligos was predicted using the Mfold web server. Then, the predicted dot bracket notation of DNA oligos was submitted to the RNAcomposer server for generation of RNA tertiary structure. It was subsequently converted to a DNA-based structure in Discovery Studio 3.5 by substitution of ribose sugar backbone and uracil residues with deoxyribose and thymine, respectively. Following geometry optimization with Hyperchem 8.0.8, molecular docking analysis was performed in AutoDock Vina employing the optimized DNA tertiary structure as receptor and the retrieved ZEN structure from PubChem as ligand.

### 2.5. Optimization of Label-Free Chemiluminescence Aptasensor

Optimization of the chemiluminescence aptasensor was performed based on a number of selected parameters, including truncated ZEN aptamers, ZEN binding temperature (4, 25 and 37 °C), binding time (5, 15 and 30 min), and ratios of DNAzyme–aptamer conjugate to the auxiliary DNA (2.5:1, 1.4:1, 1:1 and 1:1.2). For the sake of comparison, all the results were presented as the relative luminescence ratio to control.

### 2.6. Detection Specificity of the Label-Free Chemiluminescence Aptasensor

To validate the detection specificity of our chemiluminescence aptasensor, various concurring mycotoxins in foods such as AFB_1_, OTA and DON together with ZEN at a concentration of 10 ng/mL were compared.

### 2.7. Efficacy of Label-Free Chemiluminescence Aptasensor for Real Cereal Samples

To evaluate the efficacy of our proposed sensing system for real cereal samples, maize and wheat kernels were used as real food samples, spiked with 10 μg/kg ZEN and dried in the darkness. Subsequently, ZEN was extracted from corn and wheat samples using a water-based mixture of methanol (20%) and 1× PBS (80%). The extraction was performed on a shaker for 5 min. After centrifugation at 5000 r/min for 5 min, the supernatant was collected by filtering through a 0.22 μM syringe filter. The ZEN concentration in the filtered solution was measured with our chemiluminescence aptasensor. The recovery rate was calculated using the following formula: recovery rate = detected concentration/spiked concentration × 100%.

### 2.8. Data Analysis

All the experiments were conducted in triplicate. The chemiluminescence intensity was calculated to relative luminescence ratio in arbitrary units (a.u.) to control, unless otherwise stated. The result was presented as the mean of three measurements with standard deviation as the error bar. Statistical analysis of the data was carried out in Excel 2016 with the add-ins of XL-Toolbox and Analysis ToolPak. Significant differences among multiple groups were determined with a one-way analysis of variance (ANOVA) followed by the testing procedure of Fisher’s least significant difference.

## 3. Results and Discussion

### 3.1. Detection Principle of the Label-Free Chemiluminescence Aptasensor

**Figure 1:**
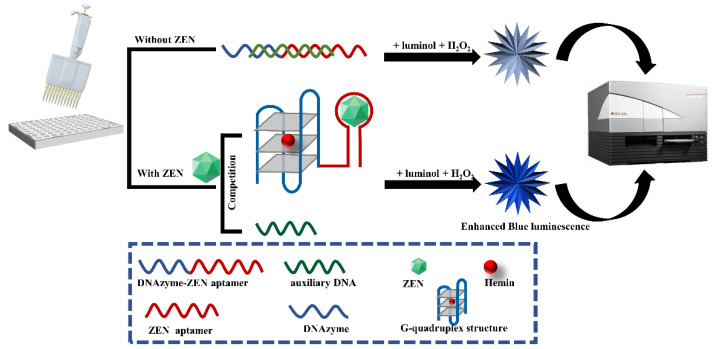
Schematic diagram of label-free and chemiluminescence detection of ZEN based on a DNAzyme–aptamer conjugate. In the absence of ZEN, DNAzyme–ZEN aptamer forms a double helix with the auxiliary DNA, leading to inactivity of the DNAzyme and subsequent weak luminescence emission upon luminol and H_2_O_2_ addition. In the presence of ZEN, it can displace the aptamer from the double helix of the DNAzyme–ZEN aptamer and the auxiliary DNA, resulting in binding of the DNAzyme to hemin and subsequent strong luminescence generation upon luminol and H_2_O_2_ addition.

The label-free chemiluminescence aptasensor for ZEN detection is constructed based on the competitive binding between ZEN analyte and an auxiliary DNA for an aptazyme, a DNAzyme conjugating to a ZEN aptamer spaced by triple adenines. As illustrated in Figure 1, in the absence of ZEN, hybridization of the auxiliary DNA to the aptazyme leads to the complete or partial blocking of the formation of G-quadruplex/hemin DNAzyme. However, in the presence of ZEN analyte, the stronger binding affinity between ZEN and the aptazyme induces activation of the G-quadruplex/hemin DNAzyme. Due to the peroxidase-like activity of this DNAzyme, it can catalyze the oxidation of luminol with H_2_O_2_ as mediator and monovalent positive ions such as K^+^ as an enhancer to generate chemiluminescence. The intensity of chemiluminescence is in direct proportion to the concentration of ZEN analyte within a certain range.

### 3.2. Feasibility of the Chemiluminescence Aptasensor

Following the detection principle, we constructed the aptasensor. Various ZEN binding buffers as well as hemin concentrations were employed for the feasibility test. As shown in Figure S1A, 1× PBS buffer supplemented with 2.5 mM MgCl_2_ and 5 mM KCl was the most feasible buffer. It gave rise to a linear trendline with R^2^ of 0.9824 within the concentration range of 1–100 ng/mL, while other the buffers—10× PBS (pH 7.4), 100 mM Tris-HCl (pH 7.4) and 100 mM Tris-HCl (pH 9.0)—resulted in poor linearity. This suggested that proper ionic strength rather than pH was the critical factor that determined the feasibility of our chemiluminescence aptasensor. Similarly, the hemin concentration also affected the feasibility of the chemiluminescence aptasensor (Figure S1B). When the hemin concentration increased from 0.5 μM to 500 μM, the linearity was greatly improved in the presence of 1–100 ng/mL ZEN. Thus, 500 μM was taken as the optimal concentration for our aptasensor. Hemin can strongly bind to the G-quadruplex, forming a peroxidase-like DNAzyme [40]. Its concentration within a certain range is proportional to the amount of active DNAzyme, which determines the luminescence signal propagation as well as the feasibility of the chemiluminescence aptasensor. This was confirmed in Figure S1C, in which the increase in luminescence intensity was positively correlated with the hemin concentration. In summary, under the condition of 1× PBS buffer and 500 μM hemin, our proposed chemiluminescence aptasensor could function properly.

### 3.3. Optimization of Experimental Parameters

**Figure 2:**
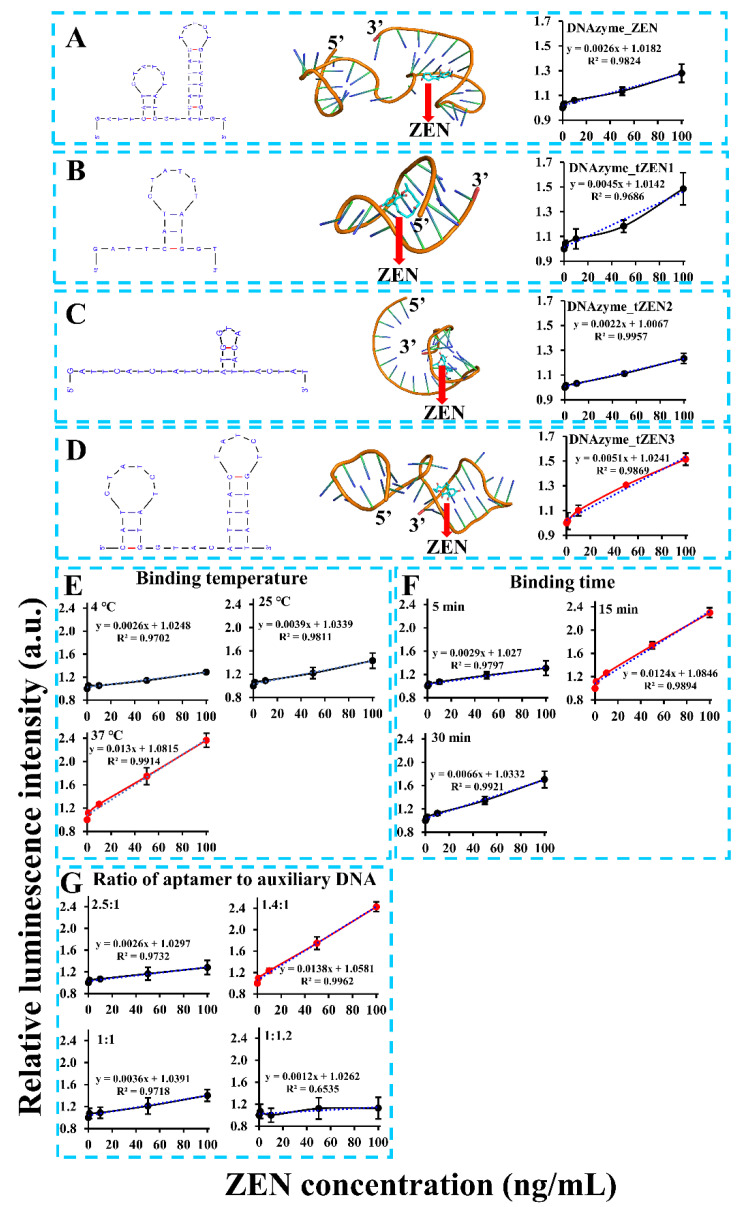
Optimization of critical parameters such as truncated ZEN aptamers (**A**–**D**), binding temperature (**E**), binding time (**F**), ratio of aptamer to auxiliary DNA (**G**) and length of auxiliary DNA.

Despite the aptasensor’s feasibility in ZEN detection, its detection sensitivity was still unsatisfactory, with a calculated limit of detection (LOD) of 20.22 ng/mL (or 63.51 nM). To address this problem, numerous factors that might affect detection sensitivity were optimized. One of the most important factors was the aptamer’s binding affinity. To remove the nonfunctional region of the aptamer, truncation of the aptamer was based on a molecular docking analysis of its binding region with ZEN. As demonstrated in Figure 2A, binding of ZEN occurred in the stem region of the second stem-loop structure via both hydrogen bonding and close contact. To validate this, we truncated the aptamer to form three versions, with the first version DNAzyme_tZEN1 leaving only the first stem-loop structure (Figure 2B), the second version DNAzyme_tZEN2 deleting nucleotides starting from the middle loop of the second stem-loop structure (Figure 2C) and the third version DNAzyme_tZEN3 maintaining both stem-loop structures but eliminating the flanking regions from both ends (Figure 2D). As a result, the aptasensor based on DNAzyme_tZEN1 displayed improved sensitivity as judged from the higher slope value (0.0045) in the linear regression function. Yet, its binding affinity to ZEN proved to be non-specific (Figure S2A). In comparison, the aptasensor based on DNAzyme_tZEN2 completely lost its original secondary structure, forming a new stem-loop achieving an even lower detection sensitivity (Figure 2C). Expectedly, it showed cross reactivity with other mycotoxins (Figure S2B). As for the aptasensor based on DNAzyme_tZEN3 and auxiliary DNA3, its enhancement in detection sensitivity was the most significant, reaching a slope value of 0.0051 (Figure 2D). Even though the structure change from DNAzyme_ZEN to DNAzyme_ZEN3 was slight, an approximately two times increase in slope value was found in the system generated from DNAzyme_ZEN3. This might be caused by the phenomenon that the truncation of flanking regions reduced non-specific binding, thereby enhancing the specific interaction between the aptamer and the target [31]. A second explanation might be that the truncated aptamer DNAzyme_ZEN3 in combination with a shorter auxiliary DNA3 increased the displacement possibility by ZEN–aptamer binding and subsequent higher detection sensitivity. The optimization of biosensor performance based on site-directed mutagenesis or rational truncation assisted by molecular docking analysis has been demonstrated to be an effective strategy [32,33,49,50]. Many studies have demonstrated that truncation of aptamers in the non-functional stem regions can achieve comparable or even significantly higher binding affinity of aptamers [21,32]. This is in agreement with our finding that truncation of the redundant stem region instead of the loop region in DNAzyme_ZEN improved the biosensor’s performance. Furthermore, the chemical synthesis cost of the aptamer could be lowered as a result of the truncation. Even though truncation of the stem region in aptamers is effective, maintaining part of the stem region is necessary for correct formation of the stem-loop structure in some cases [51]. Taken together, DNAzyme_tZEN3 preserved the backbone structure and the aptasensor built from it obtained enhanced detection sensitivity.

The second factor examined was the binding temperature between the ZEN aptamer and ZEN. It was found that incubation at 37 °C lead to a drastic improvement in detection sensitivity as visualized by the steepest slope (0.013), 2.7 and five times higher, respectively, than that of incubation at 25 °C and 4 °C (Figure 2E). Even though the original aptamer was identified under room temperature of 25 °C via SELEX strategy [46], its binding affinity towards ZEN might vary with different analytical methods. For example, Wu et al. [52] and Sun and Xie [53] reported optimal binding temperatures of 30 °C and 37 °C based on the same aptamer for detection of ZEN, respectively. To summarize, a 37 °C binding temperature was the optimal condition for our chemiluminescence aptasensor.

Other factors such as binding time and the ratio of the aptamer to the auxiliary DNA were also probed for their effect on the detection sensitivity. However, both of them made no significant improvement from their original condition. Specifically, among the tested ZEN binding times of 5, 15 and 30 min, 15 min of incubation was the optimal time, with shorter and longer binding times degrading the efficiency of aptamer’s binding affinity to ZEN (Figure 2F). This demonstrated a dynamic balance in the competition between ZEN analyte and the auxiliary DNA for the aptamer. A similar pattern was found for the ratio of the aptamer to the auxiliary DNA. The detection sensitivity was the best when the ratio was 1.4:1 (Figure 2G). Increasing or decreasing the ratio by changing the amount of auxiliary DNA significantly compromised the performance of the aptasensor. This suggested that the auxiliary DNA was instrumental in the competitive binding of ZEN to the aptamer. Even though both parameters did play a role in the aptasensor’s performance, no further enhancement in sensitivity under our tested conditions was recorded. Thus, a binding time of 15 min and 1.4:1 as the ratio of aptamer to auxiliary DNA were maintained as the optimal sensing parameters.

**Figure 3:**
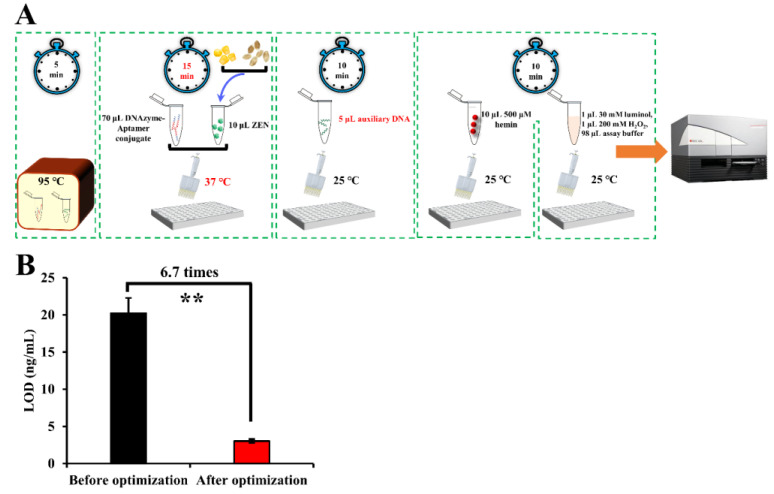
Schematic representation of the detection procedures (**A**) and improved LOD (**B**) of our proposed sensing system after the optimization process. Note: “**” denotes a very significant difference (*p* < 0.01) after Student’s *t*-test.

After optimization, 15 min of incubation time at 37 °C for ZEN binding in combination with 5 μL auxiliary DNA (namely, 1.4:1 as the ratio of the aptamer to the auxiliary DNA) was adopted as the optimal parameters for ZEN detection (Figure 3A). The LOD of our aptasensor was lowered 6.7 times as compared that before optimization (Figure 3B).

### 3.4. Analytical Performance

**Figure 4:**
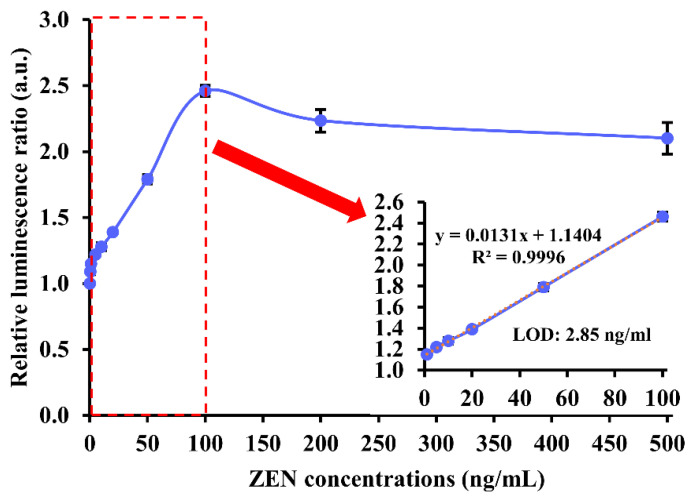
Analytical performance of our label-free chemiluminescence aptasensor in the presence of 0–500 ng/mL of ZEN target. The red dashed rectangle in the graph indicates the linear detection range of our aptasensor. The red arrow points to a magnifying graph showing the linear detection range of ZEN in 1–100 ng/mL and LOD of 2.85 ng/mL (or 8.95 nM).

To find out the analytical performance of our chemiluminescence aptasensor upon parameter optimization, 0–500 ng/mL of ZEN was used for analysis. The relative luminescence ratio experienced a steep rise in the presence of 0 to 1 ng/mL of ZEN, followed by a gradual and linear growth to around 2.5 when ZEN concentration was between 1 and 100 ng/mL. When ZEN concentration was higher than 100 ng/mL, the luminescence ratio began to descend slightly and stabilize at approximately 2.2. Hence, the detection range of the chemiluminescence aptasensor was 1–100 ng/mL (or 3.14–314.10 nM). Based on the linear range, the calculated LOD was 2.85 ng/mL (or 8.95 nM) (Figure 4).

It is not surprising that our method is inferior to the gold-standard detection technique LC-MS in every aspect [54]. However, as compared to other rapid analytical methods for ZEN, our chemiluminescence aptasensor is satisfactory in both detection range and limit. Even though some analytical methods based on electrochemical [55], fluorescent [56,57], colorimetric [58] and chemiluminescent [59] signal transduction display ultra-sensitivity of below 1 ng/mL, their detection range is much narrower as compared to our developed aptasensor (Table 1). As can also be noticed, our aptasensor is comparable in both detection range and LOD to ELISA [60], AuNPs-based lateral flow assay [52] and colorimetry [61]. To conclude, our developed aptasensor has a desirable analytical performance in comparison with other methods for ZEN detection.

### 3.5. Detection Specificity

**Figure 5:**
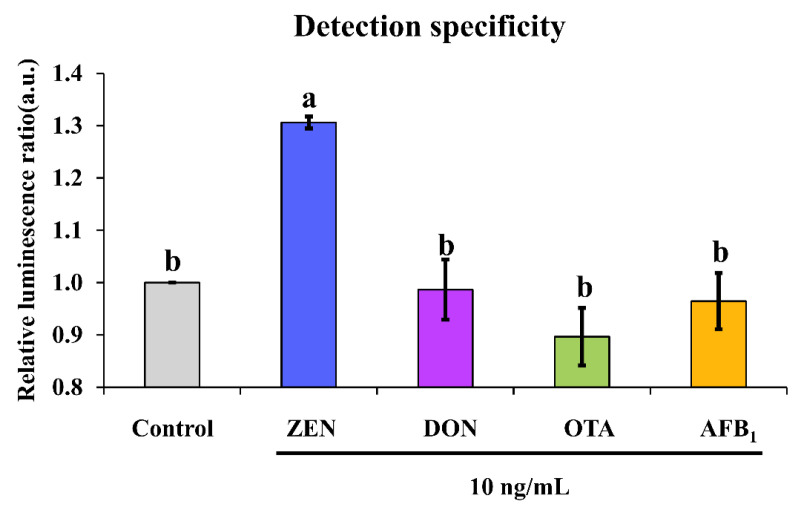
Detection specificity of our proposed sensing system. Note: different letters above bars indicate a significant difference (*p* < 0.05) after one-way ANOVA followed by Fisher’s least significant difference test.

Except for examination of the analytical performance of an aptasensor, it is more important to evaluate its cross-reactivity against other common mycotoxins that can co-contaminate foodstuffs in real-life situations. It was found that non-target mycotoxins such as DON, OTA and AFB_1_ could not induce significant chemiluminescence enhancement as ZEN did, as demonstrated by their triggered ratio of approximately 1 in comparison with a ratio of about 1.3 caused by ZEN (Figure 5). This suggested no cross-reactivity was found for our chemiluminescence aptasensor.

### 3.6. ZEN Detection in Real Samples

Following validation of the excellent sensing capability of our chemiluminescence aptasensor, its efficacy to recover ZEN from unprocessed maize and wheat kernel samples was assessed. We employed a water-based extraction buffer containing 20% methanol and 80% 1× PBS buffer to reclaim ZEN from cereal samples. Before this, no interfering effect from 20% methanol was validated on our sensing system, as suggested by the comparable slope values in two systems employing 1× PBS and 1× PBS (containing 20% methanol) as binding buffers (Figure S3). The use of 20% methanol in the extraction buffer not only has been confirmed to be efficient for mycotoxin recovery from cereal samples, but also minimizes the interfering effect of methanol on the sensing system [62,63]. To be specific on the recovery rate, we recovered 10.46 ± 1.97 and 52.50 ± 7.84 μg/kg of ZEN from 10 and 50 μg/kg ZEN-spiked maize kernel samples, achieving recovery rates of 104.57 ± 19.67% and 105.00 ± 15.68%, respectively (Table 2). A similar pattern was documented for ZEN-spiked wheat kernel samples from which 12.85 ± 0.88 μg/kg and 53.11 ± 3.92 μg/kg of ZEN was extracted for the spiked concentrations of 10 and 50 μg/kg, respectively (Table 2). This amounted to recovery rates of 128.46 ± 8.82% and 100.69 ± 7.85%, respectively. The slightly higher than 100% recovery rates for all the tested samples might suggest that there was still weak interference from the matrix. The interfering substances are probably the antioxidant substances or pigments from maize and wheat kernels, which can have an effect on the H_2_O_2_-mediated chemiluminescence reaction [64]. Overall, the good recovery capability of our chemiluminescence aptasensor from maize and wheat kernels demonstrated the excellent efficacy of our extraction buffer and negligible matrix interference with our sensing procedures. The successful recovery of 10 and 50 ng/mL ZEN meets the maximal allowable limit of 60 μg/kg in China and 20 μg/kg in the European Union for cereal products [65].

## 4. Conclusions

In conclusion, we developed a label-free chemiluminescence-based aptasensor for ZEN detection based on a conjugate of a peroxidase-like G-quadruplex DNAzyme with a truncated aptamer. This aptasensor enables rapid detection of ZEN within 40 min. In addition, it achieves a desirable detection limit of 2.85 ng/mL (or 8.95 nM) in the linear range of 1–100 ng/mL (or 3.14–314.10 nM) following optimization of the key parameters, particularly the binding temperature and the truncated aptamer. Most importantly, our proposed aptasensor is validated for its excellent specificity against various concurrent mycotoxins as well as its desirable ZEN recovery rate from realistic unprocessed cereal samples at spiked concentrations of 10 and 50 μg/kg. The chemiluminescence aptasensor can also be used for onsite and real-time determination of ZEN by integration with a portable smartphone-based detection platform with the aid of 3D printing technologies and coupled smartphone application. Owing to its low cost, satisfactory sensitivity, high specificity, short assay time and decent recovery rate, our proposed chemiluminescence aptasensor has great application potential to be employed for rapid screening of ZEN contamination in foodstuffs.

## Figures and Tables

**Table 1:** Comparison of our chemiluminescence aptasensor with other reported analytical methods.

Analytical Methods	Linear Range (ng/mL)	LOD (ng/mL)	References
ELISA	13.64–104.48	2.58	[60]
Electrochemical	1 × 10^−5^–10	5 × 10^−3^	[55]
LC-MS	1–1000	1	[54]
AuNPs-based lateral flow	5–200	20	[52]
Fluorescence	0.5–64	0.5	[56]
Fluorescence	0–10	0.8	[57]
Colorimetry	10–250	10	[61]
Colorimetry	2.5–100	0.98	[58]
Chemiluminescence	0.03–2.5	0.01	[59]
Chemiluminescence	1–100	2.85	this work

**Table 2:** Recovery rate for ZEN-spiked real maize and wheat samples.

Real Samples	Spiked Concentration (μg/kg)	Detected Concentration (μg/kg)	Recovery Rate (%)
**maize**	10	10.46 ± 1.97	104.57 ± 19.67
	50	52.50 ±7.84	105.00 ± 15.68
**wheat**	10	12.85 ± 0.88	128.46 ± 8.82
	50	53.11 ± 3.92	100.69 ± 7.85

## Data Availability

The data presented in this study are available on request from the corresponding author.

## Supplementary Materials

The following supporting information can be downloaded at: https://www.mdpi.com/article/10.3390/bios13010118/s1. Table S1: DNA oligos used in this study.; Figure S1: Feasibility exploration of label-free and chemiluminescence detection of ZEN based on a DNAzyme–aptamer conjugate by selection of various binding buffers (A) and hemin concentrations (B and C); Figure S2: Detection specificity of the chemiluminescence aptasensor based on truncated aptamers: DNAzyme_tZEN1 (A) and DNAzyme_tZEN2 (B). Figure S3. Effect of 20% methanol in 1× PBS binding buffer (B) on the performance of the chemiluminescence aptasensor as compared to 1× PBS control buffer (A).
