# Supplementary material for: A Novel and Label-Free Chemiluminescence Detection of Zearalenone Based on a Truncated Aptamer Conjugated with a G-Quadruplex DNAzyme
Source: Biosensors (Basel). 2023 Jan 9;13(1):118. doi: 10.3390/bios13010118 (PMC9856370; doi:10.3390/bios13010118)
Supplement: Supplementary file 1 [file biosensors-13-00118-s001.zip › biosensors-2117454-supplementary.pdf]

Article

A Novel and Label-Free Chemiluminescence Detection of Zearalenone Based on a Truncated Aptamer Conjugated with a G-Quadruplex DNAzyme

Yue Guan [1,2,†], Junning Ma [3,4,†], Jing Neng [1,2], Bolei Yang [3,4], Yan Wang [1,2,*] and Fuguo Xing [3,4,*]

1 College of Food Science and Technology, Zhejiang University of Technology, Hangzhou 310014, China
2 Key Laboratory of Food Macromolecular Resources Processing Technology Research, Zhejiang University of Technology, China National Light Industry, Hangzhou 310014, China
3 Key Laboratory of Agro-Products Quality and Safety Control in Storage and Transport Process, Ministry of Agriculture and Rural Affairs, Beijing 100193, China
4 Institute of Food Science and Technology, Chinese Academy of Agricultural Sciences, Beijing 100193, China
* Correspondence: wangyan062006@zjut.edu.cn (Y.W.); xingfuguo@caas.cn (F.X.)
† These authors contributed equally to this work.

Table S1. DNA oligos used in this study.

Oligo names	Sequences
DNAzyme_ZEN	TGGGTAGGGCGGGTTGGGAAAGATTCATCTATCTATGGTACATTACTATCTGTAATGTGA
DNAzyme_tZEN1	TGGGTAGGGCGGGTTGGGAAAGATTCATCTATCTATGGT
DNAzyme_tZEN2	TGGGTAGGGCGGGTTGGGAAAGATTCATCTATCTATGGTACATTACTAT
auxiliary DNA	AGTAATGTACCATAGATAGATGAATCTTTCCCAACCCGCC
DNAzyme_tZEN3	TGGGTAGGGCGGGTTGGGAAACATCTATCTATGGTACATTACTATCTGTAAT
auxiliary DNA3	AGTAATGTACCATAGATAGATGTTTCCCAACCCGCC

Note: red-colored letters represent DNAzyme or reverse-complement counterpart, while blue-colored letters denote ZEN aptamer or reverse-complement counterpart. Underlined letters indicate hybridized locations between DNAzyme-ZEN aptamer conjugate and the auxiliary DNA.

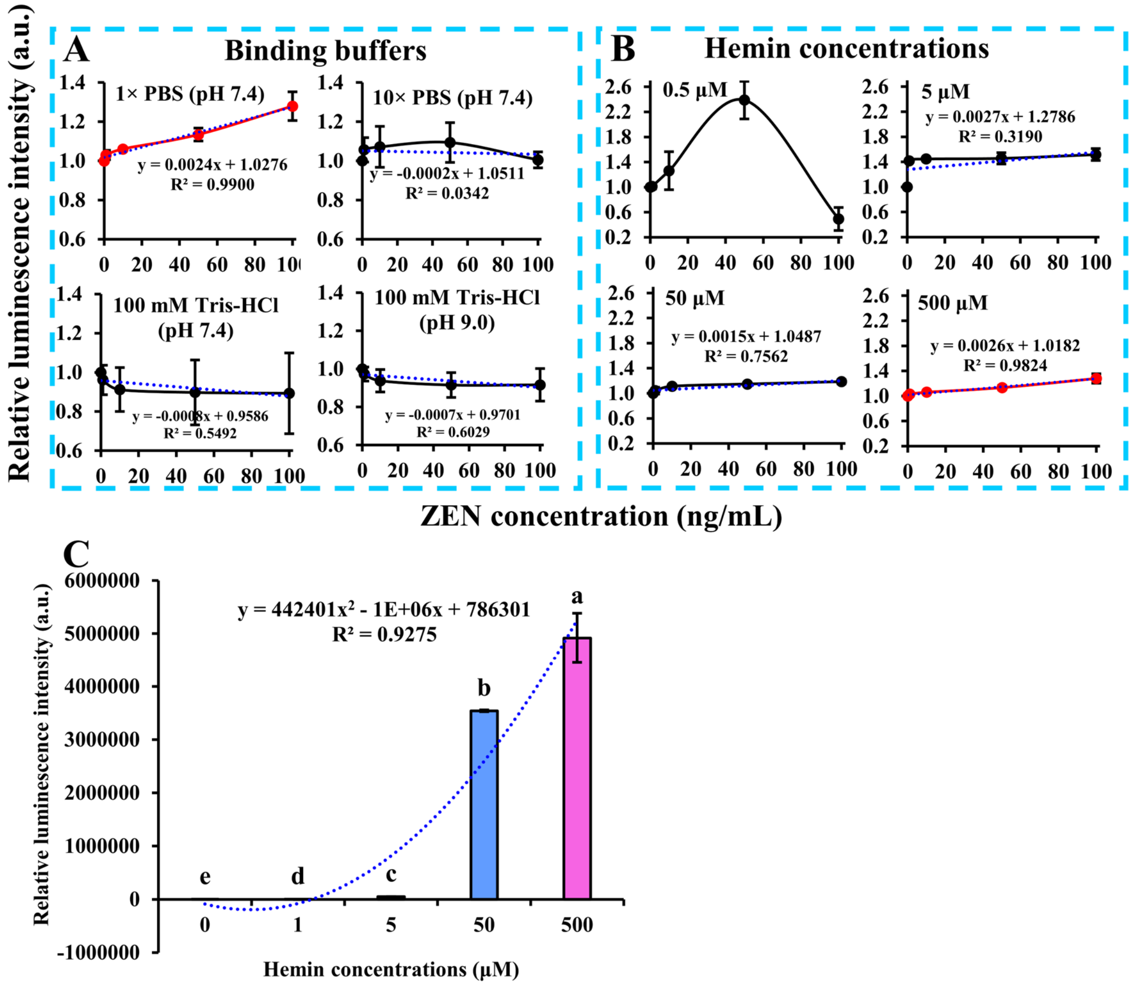

Figure S1. Feasibility exploration of label-free and chemiluminescence detection of ZEN based on a DNAzyme-aptamer conjugate by selection of various binding buffers (**A**) and hemin concentrations (**B** and **C**). Note: different letters denote significant difference ($p < 0.05$) determined by one-way ANOVA followed by Fisher's least significant difference.

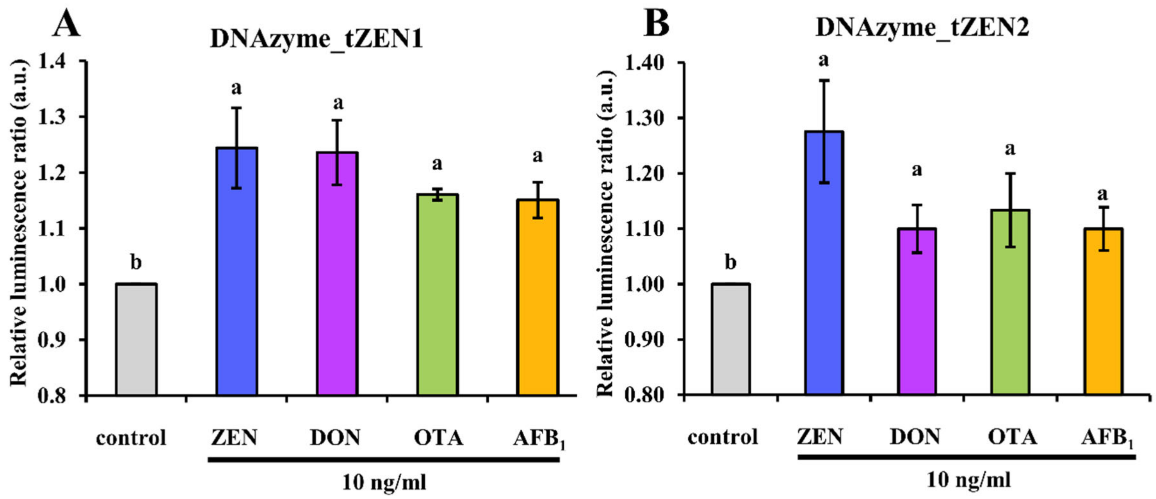

Figure S2. Detection specificity of the chemiluminescence aptasenor based on truncated aptamers: DNAzyme_tZEN1 (**A**) and DNAzyme_tZEN2 (**B**). Note: different letters denote significant difference ($p < 0.05$) determined by one-way ANOVA followed by Fisher's least significant difference.

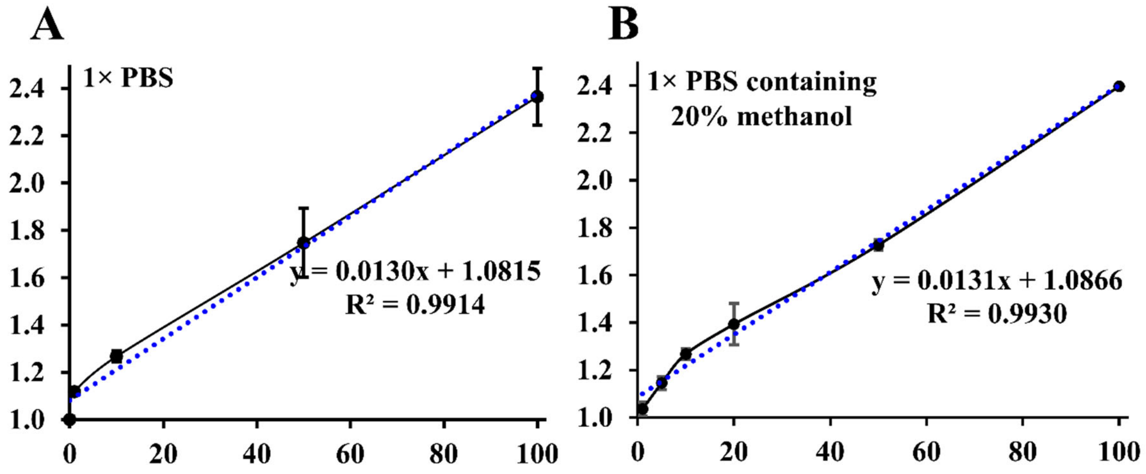

Figure S3. Effect of 20% methanol in 1× PBS binding buffer (**B**) on the performance of the chemiluminescence aptasenor as compared to 1× PBS control buffer (**A**).
